# Time-series transcriptomic screening of factors contributing to the cross-tolerance to UV radiation and anhydrobiosis in tardigrades

**DOI:** 10.1186/s12864-022-08642-1

**Published:** 2022-05-28

**Authors:** Yuki Yoshida, Tadashi Satoh, Chise Ota, Sae Tanaka, Daiki D. Horikawa, Masaru Tomita, Koichi Kato, Kazuharu Arakawa

**Affiliations:** 1 Institute for Advanced Biosciences, Keio University, Nihonkoku, 403-1, Daihouji, Tsuruoka, Yamagata 997-0017 Japan; 2 Systems Biology Program, Graduate School of Media and Governance, Keio University, 5322 Endo, Fujisawa, Kanagawa 252-0882 Japan; 3 Faculty and Graduate School of Pharmaceutical Sciences, Nagoya City University, 3-1 Tanabe-dori, Mizuho, Nagoya 467-8603 Japan; 4 Exploratory Research Center On Life and Living Systems (ExCELLS), National Institute of Natural Sciences, 5-1 Higashiyama, Myodaiji, Okazaki, Aichi 444-8787 Japan

**Keywords:** Tardigrade, Anhydrobiosis, Ultraviolet C, Antioxidative stress

## Abstract

**Background:**

Tardigrades are microscopic animals that are capable of tolerating extreme environments by entering a desiccated state of suspended animation known as anhydrobiosis. While antioxidative stress proteins, antiapoptotic pathways and tardigrade-specific intrinsically disordered proteins have been implicated in the anhydrobiotic machinery, conservation of these mechanisms is not universal within the phylum Tardigrada, suggesting the existence of overlooked components.

**Results:**

Here, we show that a novel Mn-dependent peroxidase is an important factor in tardigrade anhydrobiosis. Through time-series transcriptome analysis of *Ramazzottius varieornatus* specimens exposed to ultraviolet light and comparison with anhydrobiosis entry, we first identified several novel gene families without similarity to existing sequences that are induced rapidly after stress exposure. Among these, a single gene family with multiple orthologs that is highly conserved within the phylum Tardigrada and enhances oxidative stress tolerance when expressed in human cells was identified. Crystallographic study of this protein suggested Zn or Mn binding at the active site, and we further confirmed that this protein has Mn-dependent peroxidase activity in vitro.

**Conclusions:**

Our results demonstrated novel mechanisms for coping with oxidative stress that may be a fundamental mechanism of anhydrobiosis in tardigrades. Furthermore, localization of these sets of proteins mainly in the Golgi apparatus suggests an indispensable role of the Golgi stress response in desiccation tolerance.

**Supplementary Information:**

The online version contains supplementary material available at 10.1186/s12864-022-08642-1.

## Background

Tardigrades are microscopic animals that are renowned for the ability of terrestrial species to enter a state of suspended animation known as cryptobiosis [1], or more particularly, anhydrobiosis (life without water), which is cryptobiosis upon almost complete desiccation. Tardigrades can withstand extreme conditions in this dormant state (several in the active state as well), including extreme temperature, pressure, high doses of ionizing radiation and exposure to the vacuum of space [2–8], yet they quickly resume life within the hour upon rehydration. Anhydrobiosis has been acquired in multiple lineages encompassing all kingdoms of life, but tardigrades are unique in multi-cellular animals that terrestrial and limno-terrestrial species can enter anhydrobiosis [9], and that the anhydrobiosis in tardigrades generally does not rely on trehalose [10–12]. The molecular machinery of tardigrade anhydrobiosis is beginning to be uncovered due to the availability of genomic resources [11, 13–22]. Tardigrades employ both elements highly conserved within eukaryotes and lineage-specific elements for anhydrobiosis. The above genome and transcriptome analyses suggested common adaptations of highly conserved anhydrobiosis genes such as anti-oxidative stress proteins (superoxide dismutase, glutathione, thioredoxin, and catalase), chaperones (heat shock proteins), DNA repair enzymes (RAD51), etc. [23–27], and has also led to the identification of several tardigrade-specific genes encoding for proteins such as CAHS, SAHS, MAHS, LEAM, and Dsup, that have been suggested to play critical roles in cellular protection upon anhydrobiosis [14, 28–32]. Notably, Dsup is a nucleus-localizing DNA-binding protein that is reported to protect DNA molecules from hydroxyl radicals, where the induction of this single protein in mammalian cells and plants can increase its radiation tolerance [14, 33–35]. However, these proteins have been acquired after the divergence of the class Eutardigrada (as they are not present in the other class, Heterotardigrada) [15, 16], thus the necessary and sufficient set of genes and pathways enabling anhydrobiosis remains elusive. These observations led to the hypothesis that a tardigrade-specific, but conserved widely in Tardigrada, anhydrobiosis protein family may exist.

Of the many adverse extremes tardigrades can tolerate in anhydrobiosis, radiation is unique in that tardigrades can tolerate it in both the active hydrated state and the inactive desiccated state [36, 37], suggesting the existence of efficient repair pathways in addition to the protective mechanisms identified thus far. Tolerance to radiation in tardigrades is a cross-tolerance of anhydrobiosis [38], and the overlapping pathway is presumably the defense against reactive oxygen species (ROS) that mediates protein oxidation and DNA damage [39, 40]. To this end, we employed ultraviolet C (UVC), a low-level energy stressor that causes oxidative stress, to screen for tardigrade-unique components for ROS defense in *Ramazzottius varieornatus* Bertolani & Kinchin, 1993. Tardigrades are capable of tolerating approximately 1,000-fold higher dosages of ultraviolet B (UVB) and UVC than human cell lines [6, 41].

## Results

### Cellular responses against UVC exposure and comparison with desiccation entry

**Fig. 1:**
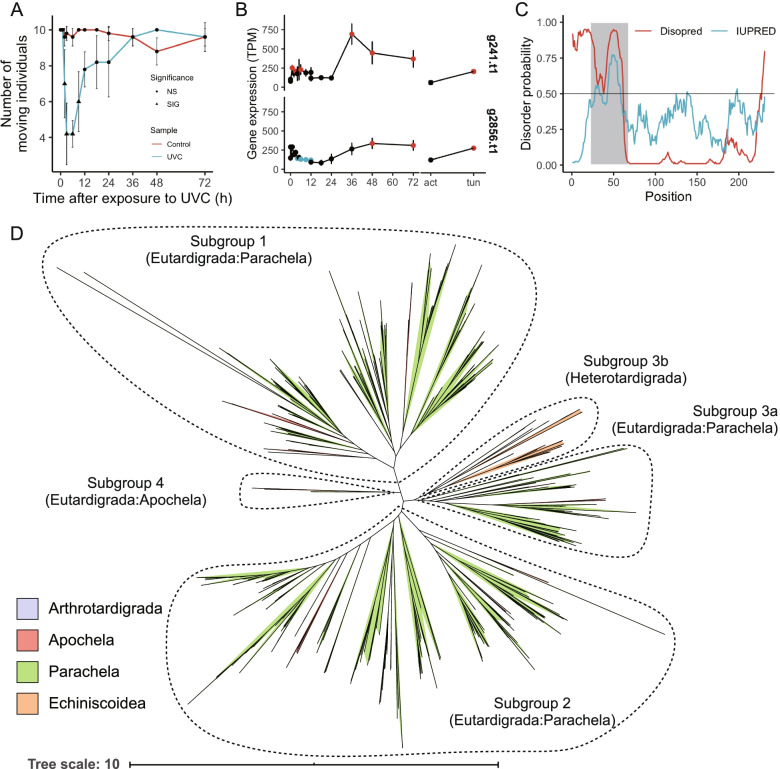
Identification of a novel stress responsive gene family conserved within phylum Tardigrada. **a** *R. varieornatus* specimens were exposed to 2.5 kJ/m^2^ of UVC and movements were observed at each time point. Significant differences between samples are indicated as a triangle in the UVC sample time course (Tukey HSD test, Adjusted *p*-value < 0.05). Error bars indicate standard deviation. **b** Expression values of two tardigrade-specific genes with no known annotations. Error bars indicate standard deviation. Colors indicate significant expression changes; blue: significantly decreased, red: significantly increased, black: non-significant change. **c** Probability plot of disordered regions in the g12777 protein predicted by DISOPRED or IUPRED. Disordered regions predicted are indicated with a gray highlight. **d** Phylogenetic tree of OG0000231 orthologs in Tardigrada. Each ortholog was colored by the corresponding lineage. Four subfamilies were found and named according to the major lineage

Previous studies have observed that *R. varieornatus* exposed to 2.5 kJ/m^2^ UVC showed a prolonged decrease in the movement for approximately 1 day [6], which presumably is the critical period for the ROS response. We first validated this observation on a finer time scale, where individuals who were exposed to 2.5 kJ/m^2^ showed significantly lower movements from 2–9 h after exposure (Fig. 1a). This suggests that cellular repair may be occurring during this period, relatively consistent with our previous studies analyzing the expression of the photorepair gene *phrA*. However, the duration of cellular repair and the extent of regulation remains to be unknown, thus we conducted transcriptome profiling from 0–12 h and 0–72 h to screen for genes induced in this period (Table S1). Initial clustering of expression values using Spearman correlation indicated that the transcriptome profiles drastically shifted between 3–4- and 24–36-h post-exposure (Additional file 1: Figure S1), suggesting consistency with the motility of the animals. We found a total of 3,324 differentially expressed genes following exposure to UVC (DESeq2, FDR < 0.05), of which 1,314 and 2,110 genes to be up-regulated and down-regulated, accordingly (Additional file 1: Figure S1). Genes with high fold change (> 4) were comprised of various genes, including chaperones (Mitochondrial chaperone BCS1), DNA damage repair pathways (XRCC4, PARP), metalloproteases (NAS-13), anti-oxidative pathways (GST), and previously identified tardigrade specific protection-related genes (CAHS and SAHS). Interestingly, DEGs that had high expression values included several of those with high fold change, additionally anti-oxidative stress genes thioredoxin and peroxiredoxin (Additional file 2: Table S2, Additional file 2: Table S3). These genes were induced during tardigrade anhydrobiosis [11], suggesting similar pathways are being regulated between desiccation and UVC exposure. Additionally, we found that the zinc metalloprotease NAS, apolipoproteins, autophagy-related sequestosome, and the mitochondrial chaperone BCS1 were highly expressed (TPM > 1,000) after exposure. We have found similar inductions of sequestosome and BCS1 during *Hypsibius exemplaris* Gąsiorek et al. 2018 anhydrobiosis [11], suggesting the existence of mitochondria stresses from oxidative stress during desiccation or UVC exposure–response.

### Identification of a tardigrade specific gene family as a novel stress responsive gene family

To screen for genes responsible for the cross-tolerance of anhydrobiosis and UVC exposure, we analyzed the intersection of DEGs in the above result and our previous differential transcriptome analysis during slow desiccation as repair genes had been induced during anhydrobiosis entry [11]. We found 141 genes that were upregulated in both conditions (Additional file 2: Table S4, Additional file 2: Table S5). These genes were significantly enriched in Gene Ontology terms related to antioxidative stress and lipid transport (*e.g.,* glutathione transferase activity, superoxide dismutase activity, etc., Table 1). Sixty-six of these genes had Swiss-Prot homologs (BLASTP, E-value < 1E-15, Additional file 2: Table S4), which included the CAHS and SAHS genes, antioxidative stress response genes (*e.g.*, SOD, GST), and other genes that have been implied to be related to anhydrobiosis (*e.g.*, apolipoprotein, sequestosome). Other tardigrade specific anhydrobiosis genes (MAHS, RvLEAM, Dsup) were not contained in this list. Thirty-four of the genes within the remaining seventy-five were predicted to have functional domains (PANTHER, Pfam, SMART, and SUPERFAMILY domains detected by InterProScan, Additional file 2: Table S5). This included antioxidative stress-related domains (GST, eukaryotic cytochrome b561, ferritin-like domain), DNA binding domains (zinc finger, EF-hand), transmembrane proteins (tetraspanin, EXPERA, and major facilitator superfamily), extracellular binding (Cadherin_repeat, bacterial extracellular solute-binding proteins, family 3), other domains we have seen in our previous studies (zinc metalloprotease, kazal-type serine protease inhibitor domain, fatty acid-binding domain, and chitin-binding domain, and fasciclin domain).

**Table 1:** Gene ontology enrichment of differentially expressed genes common between anhydrobiosis and UVC response

GOBPID	*p*-value	Term
GO:0,006,869	0.00703419	lipid transport
GO:0,006,801	0.00865766	superoxide metabolic process
GO:0,006,749	4.03E-05	glutathione metabolic process
GO:0,006,575	8.62E-06	cellular modified amino acid metabolic process
GO:0,010,876	0.01043244	lipid localization
GO:0,072,593	0.01137521	reactive oxygen species metabolic process
GO:0,006,790	0.00041013	sulfur compound metabolic process
GO:0,051,186	5.22E-05	cofactor metabolic process
GO:0,043,603	0.01760895	cellular amide metabolic process
GO:0,006,518	0.04178322	peptide metabolic process

To validate the conservation of these genes within Tardigrada, we submitted amino acid sequences to a BLAST search against our in-house and publicly accessible genomes/transcriptomes [11, 14, 15]. We first predicted approximately 10–90 thousand genes in various tardigrade lineages and then identified orthologs by BLASTP search or OrthoFinder clustering (Additional file 2: Table S6). From the OrthoFinder clustering, we observed twenty-one genes out of the 75 genes without Swiss-Prot homologs were conserved within Metaoza (Additional file 2: Table S5**)**. Twenty of the remaining 54 were *Ramazzottius*-specific genes, where 18 did not have any InterProScan annotations. The remainder were conserved widely or in part within Eutardigrada. Within the 7 genes conserved between Heterotardigrada, we found only two genes (g2856/RvY_14843, g241/RvY_00334) had homologous genes in *E. testudo* (TBLASTX, E-value < 1e-5) [16] and with no known functional domains (InterProScan, CDD, Pfam-A, Superfamily, SMART, Fig. 1b**, **Additional file 2: Table S5**)**. Both genes belonged to a multi-copy gene family with high expression profiles (g2856 OG0000231, g241 OG0000230. Additional file 2: Table S7, Additional file 2: Table S8, Additional file 2: Table S9, Additional file 2: Table S10). Additional blastp searches identified four more clusters to be orthologous to OG0000231 (OG0001788, OG0012107, OG0024270, OG0052053, designated OG0000231 family in following text) and one more to OG0000230 (OG0005452, designated OG0000230 family in following text). The OrthoFinder results indicated most of the lineages showed conservation of OG0000231 family (excluding the Halechiniscidae family in Arthrotardigrada), indicating that this gene family is highly conserved in the phylum (Additional file 2: Table S7). On the contrary, we found that OG0000230 family was lost in the Apochela and Echiniscoididae lineages (Additional file 2: Table S8). We also have found a OG0000230 family ortholog in the arthrotardigrade Batillipes by BLAST and Orthofinder based analysis, however validation by phylogenetic analysis could not rule out the possibility of contamination, thus we suspend on concluding conservation within class Arthotardigrada. Additionally, initial TBLASTX searches against the publicly accessible *Echiniscoides sigismundi* and *Richtersius coronifer* transcriptome assemblies indicated the loss of OG0000231 orthologs in both species. However, we identified several raw RNA-Seq reads that showed homology with coding sequences of OG0000231 coding genes, which implied the existence of OG0000231 orthologs. BLASTX searches against the reassembled *E. sigismundi* and *R. coronifer* transcriptome identified approximately fifteen OG0000231 orthologs but no OG0000230 orthologs in *E. sigismundi*. On the other hand, we detected twenty-three OG0000230 and sixty-four OG0000231 orthologs in *R. coronifer*. The lack of OG0000230 or OG0000231 orthologs in previous transcriptome assemblies may have occurred during the filtering stage. Our in-house transcriptome sequencing data supported the existence of both gene families in *R. coronifer* (seventeen OG0000230 and fifty-six OG0000231 orthologs) and twelve OG0000231 orthologs in *E. sigismundi*. Together, these results support that the OG0000230 is lost in the Echiniscoididae, Halechiniscidae, and Apochela lineages. Three of the OG0000231 orthologs (including g2856) were found to be mis-predicted in our gene set (Additional file 2: Table S11).

The OG0000230 orthologs in *H. exemplaris* have been identified as a horizontally transferred gene candidate in our previous study (Additional file 2: Table S12). Phylogenetic analysis suggested that the entry of this gene into tardigrade genomes may have occurred in the early stages of the Tardigrada lineage (Additional file 1: Figure S2). BLASTP searches against TrEMBL and NCBI nr databases indicated that the majority of homologs originates from Gammaproteobacteria species (Additional file 2: Table S13, Additional file 2: Table S14). This suggests that this gene family may have been integrated into the tardigrade genome before the divergence of these Eutardigrada and Heterotardigrada. We also have found several orthologs in Ciliophora, Chlorophyta, and Acanthamoeba, but the conservation patterns suggest that these genes may also be results of horizontal transfer into these organisms. Several of these bacterial orthologs were found to be fused to the C-terminal end of haem peroxidases, however these regions do not have any functional domains and are not predicted to be a disordered region, suggesting that this gene itself may not have anti-oxidative stress functions.

The remaining OG0000231 was strongly multiplied within the *R. varieornatus* genome (total of 35 copies), and several of the orthologs were duplicated in tandem (Additional file 2: Table S7, Additional file 2: Table S15) and were highly conserved throughout the phylum Tardigrada, including in non-anhydrobiotic species of Heterotardigrada. Amino acid sequences of this protein family seem to be relatively diverse, with core conserved domain surrounded by variable domain architectures (Additional file 1: Figure S3). Phylogenetic analysis of OG0000231 orthologs indicated that there are 4 subgroups (Fig. 1d), where a single subfamily was comprised of Heterotardigrada species. Within the 35 copies of the OG0000231 gene family, the g12777 gene was found to have constant high expression (Short 225–407 TPM, Long 75–337 TPM) during the UVC exposure response, fast desiccation (Active 97.5 TPM, Tun 369.1 TPM), and 2.5-fold induction of expression during slow desiccation (active 98, Tun 238) of *R. varieornatus*. *H. exemplaris* orthologs were highly induced during anhydrobiosis entry (39 copies, median 7.0x, maximum 414x), thus implying this protein family’s relationship with anhydrobiosis. Additionally, the informatics-based analysis predicted a signal peptide and a disordered region in the N-terminus of the g12777 protein (Fig. 1c), similar to various tardigrade-specific anhydrobiosis-related genes. These characteristics suggested that this gene may play an important role in tardigrade anhydrobiosis; therefore, we submitted this g12777 gene for further functional analysis.

### g12777 has a highly conserved Mn^2+^ biding site

**Fig. 2:**
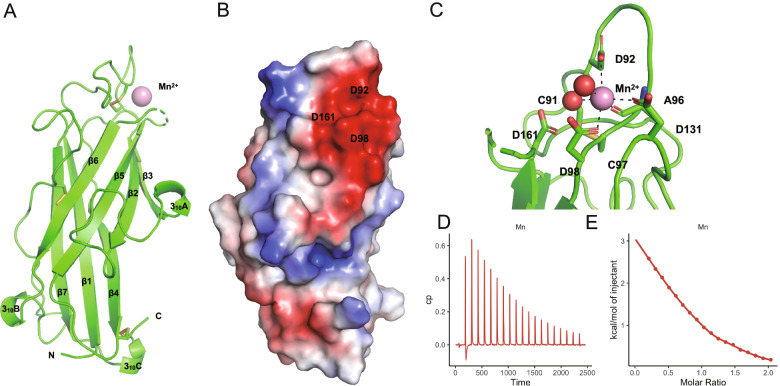
g12777 protein has a SOD-like -sandwich fold and binds to Mn^2+^ ion. **a** The crystal structure of the catalytic domain of the g12777 protein. Bound Mn^2+^ ion and residues involved in disulfide bond formation are indicated with a pink sphere and stick, respectively. Positions of the N and C termini are indicated as letters. **b** Electrostatic surface potential of g12777 protein. The surface model of g12777 is colored according to the electrostatic surface potential (blue, positive; red, negative; scale from -50 to + 50 kT/e). **c** Close-up view of the Mn^2+^-binding site. The binding site is comprised from three aspartic acid residues (D92, D98, and D131) and a disulfide bond with close proximity (C91 and C97). **d**,**e** Mn^2+^ binding affinity measured by isothermal titration calorimetry. **d** raw data in seconds **e** integrated heat values corrected for the heat of dilution and fit to a one-site binding model (solid line)

We crystallized the putative globular domain that lacked the N-terminus 62 amino acids of g12777 protein (g12777-Δ62) and solved the crystal structures as two forms containing Mn^2+^ or Zn^2+^ ions at 2.30 Å and 1.60 Å resolutions, respectively (Fig. 2a, Additional file 1: Figure S4a,b, Additional file 2: Table S16). The N-terminus 62 amino acids were removed due their disordered nature that hinders crystallization. The Zn^2+^ g12777 protein structure was found to be bound to five Zn^2+^ ions originating from the crystallization buffer containing 300 mM zinc acetate, but not to Ca^2+^ from the protein buffer (2 mM calcium chloride). The Mn^2+^-bound g12777 crystals prepared by soaking method (50 mM Mn^2+^) using Zn^2+^-bound crystals indicated that the Zn^2+^ ion in the Zn-1 site was replaced with Mn^2+^. Additionally, an Mn^2+^ ion was found to be bound at a new binding site (Mn-2, Additional file 1: Figure S4a,b), whereas the Zn^2+^ in Zn-2–5 disappeared. The Zn^2+^ crystal structure of putative catalytic domain (residues Gly63–Leu231) of the g12777 protein displayed a characteristic β-sandwich fold comprising seven β-strands and three 3_10_ helices with three disulfide bridges (C91-C97, C146-C225, and C176-C200) (Additional file 1: Figure S4b,c). A part of the β5–β6 loop (residues 163–168) around the Mn^2+^-binding site was disordered in the Mn^2+^-bound crystal (Fig. 2a), suggesting its flexible nature. Comparison of the structure of the β-sandwich domain of g12777 with known protein structures revealed that the g12777 protein structure has very weak similarities with structures of calcium-binding C2 domain involved in lipid interaction (*Z*-score = 5.1–5.5; RMSD = 2.5–3.2 Å; identity = 5–13%; PDB codes: 2JGZ, 1WFJ, and 4IHB) and subtle similarity with a Cu/Zn-SOD monomeric circular permutant (*Z*-score = 4.1; RMSD = 3.4 Å; identity = 5%; PDB code: 5J0C).

Together, the g12777 protein possesses a β-sandwich domain sharing a common Mn^2+^ and Zn^2+^ binding site. In this site, an Mn^2+^ ion was coordinated by three aspartic acids with high electrostatic surface potential (Fig. 2bc). In the Mn^2+^-binding site located at the negatively charged patch comprising β1–β2 and β3–β4 loops, the side-chain oxygen atoms of Asp92, Asp98, and Asp131 and the main-chain carbonyl atom of Ala96 and two water molecules coordinate with Mn^2+^ ion at distances of 2.2–2.9 Å (Fig. 2c). As for the Zn^2+^-binding site located at the almost same region as that of Mn^2+^, the side-chain oxygen atoms of Asp92, Asp98, Asp161, and Asp163 coordinate with Zn^2+^ ion at distances of 1.9–2.0 Å (Additional file 1: Figure S4d). A considerable conformational difference was observed between Mn^2+^- and Zn^2+^-bound forms in terms of the metal-binding site, in which Asp92 and Asp98 were commonly involved in Mn^2+^ and Zn^2+^ binding (Fig. 2c and Additional file 1: Figure S4d). These aspartic acid residues were highly conserved within tardigrade orthologs (Additional file 1: Figure S4ef), and the highly conserved CD-CD motif containing two Asp residues (D92 and D98, Additional file 1: Figure S4ef) was used in the metal ion binding in both structures, suggesting the importance of these residues. The cysteine residues in this motif (C91 and C97) formed a disulfide bond in the crystal structure, suggesting that this disulfide bond may also contribute to this protein’s function. We also observed another region that was highly conserved (W194 and R216, Additional file 1: Figure S4f), suggesting that another functional site may be present.

ITC experiment demonstrated that the estimated dissociation constants of the catalytic domain of the g12777 protein and Zn^2+^, Mn^2+^, and Ca^2+^ were 1.92 × 10^–6^ ± 6.00 × 10^–8^ M, 2.42 × 10^–5^ ± 1.75 × 10^–6^ M, or 1.39 × 10^–4^ ± 3.02 × 10^–5^ M, respectively, indicating that the binding affinity of Zn^2+^ was the highest among these divalent cations (Fig. 2de, Additional file 1: Figure S4g, Additional file 2: Table S17). Importantly, the binding stoichiometry (*n*) of Zn^2+^, Mn^2+^, and Ca^2+^ were 1.00 ± 0.00, 0.77 ± 0.02, and 0.80 ± 0.17, indicating that the catalytic domain of the g12777 protein binds to one metal ion in solution.

### g12777 is a Mn^2+^ dependent peroxidase

**Fig. 3:**
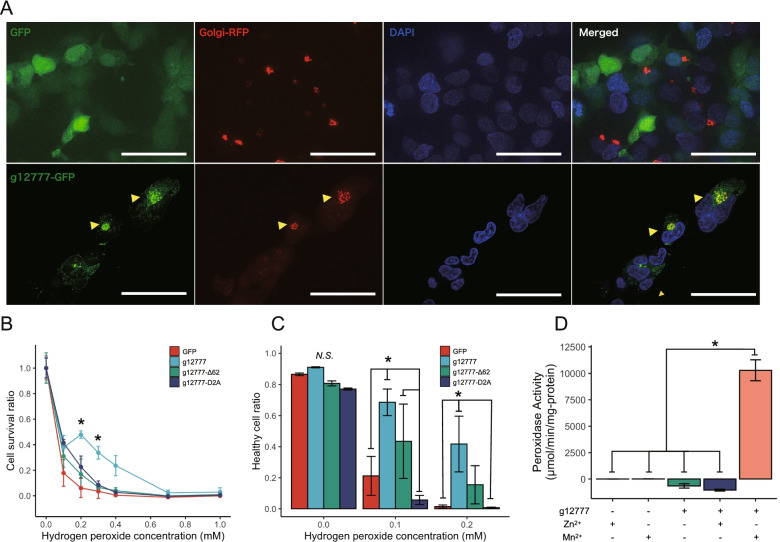
g127777 localizes in the Golgi apparatus and enhances anti-oxidative stress response in HEK293 cells. **a** The g12777 protein was fused to GFP and transfected to HEK293 cells. Cells were transfected with CellLight Golgi-RFP and stained with DAPI. Yellow arrows indicate the co-localization of g12777 and Golgi-RFP. Scale bar 25 µm. The images have been enhanced for visualization. **b** Cell transfected with g12777-GFP were exposed to hydrogen peroxide for 30 min and subjected to MTT assay after 24 h incubation. Error bars indicate standard deviation. Only the full-length g12777-GFP show an increase in survival at around 0.2–0.3 mM. ANOVA + Tukey HSD (* FDR < 0.05). **c** Cells transfected with g12777-GFP were exposed to hydrogen peroxide for 30 min and subjected to MACS flow cytometry to detect SYTOX blue and AnnexinV-Alexa 657 fluorescence after 24 h incubation. Error bars indicate standard deviation. Only full-length g12777-GFP show an increase in survival. ANOVA + Tukey HSD (* FDR < 0.05). **d** Peroxidase activity of the recombinant protein lacking the N terminal 62aa region (g12777-Δ62). Peroxidase function was present only when manganese ions were present. Error bars indicate standard errors. ANOVA + Tukey HSD (* FDR < 0.05)

To validate where this protein functions in cultured cells, we constructed a GFP-tagged recombinant g12777 protein in the pAcGFP1-N1 plasmid and expressed these proteins in the HEK293 cell line. Staining with DAPI and expression of CellLight GolgiRFP suggested that this protein localizes mainly in the Golgi apparatus (Fig. 3a). Six of the most highly expressed orthologs of the OG0000231 family (highly expressed in the UVC time course and differentially expressed during slow-dry anhydrobiosis) also showed mainly a Golgi apparatus localization (Additional file 1: Figure S5). To validate this protein’s antioxidative stress functions, we subjected cells expressing GFP-tagged g12777 proteins to oxidative stress induced by exposure to hydrogen peroxide (H_2_O_2_). Desiccation tolerance requires protection of RNA, DNA, proteins, organelles, and membranes, and UVC tolerance requires DNA damage protection/repair as well as oxidative stress response, thus both are highly multifactorial that would obscure the cause of cellular death and hinder functional analysis of this protein. The transfected cells showed increased survival at 0.2–0.3 mM H_2_O_2_ as measured by MTT assays (Fig. 3b). Similar results were obtained (0.1–0.2 mM H_2_O_2_) by flow cytometry measurements (Annexin V-negative + SYTOX Blue-negative cells, Fig. 3c). Substitution of the Asp residues comprising Zn-1 (Asp92, Asp98, Asp161, and Asp163) to alanine (g12777-D2A) showed a decrease in cell survival (Fig. 3bc), indicating that this binding site is required for antioxidative stress function. We then assayed whether recombinant proteins also have an antioxidative stress function. The recombinant β-sandwich domain of g12777 protein (g12777-Δ62) showed significant peroxidase activity when Mn^2+^ ions but not Zn^2+^ ions were present (Fig. 3d). The sample buffer used for this assay contains 0.1% BSA, and the manufacturer warrants the lack of non-specific protein catalysis by BSA up to 1 mg/ml with this kit. This reaction (H_2_O_2_ + CH_3_OH → HCHO + 2H_2_O) was carried out without the presence of hemes, thus suggesting that this protein is distinguished from most Mn peroxidases. The peroxidase activity of g12777 in Mn^2+^ conditions was 1/20 of that of bovine liver catalase (Additional file 1: Figure S6). Removal of the signal peptide and disorder region (g12777-Δ62) did not increase tolerance against oxidative stress (Fig. 3bc), suggesting that the localization into the Golgi apparatus is required.

## Discussion

In this study, we have conducted transcriptome analysis of *R. varieornatus* exposed to UVC exposure, and through comparison with the transcriptome profiles during anhydrobiosis entry, have identified a novel protein family with peroxidase activity. Anhydrobiosis and desiccation tolerance is achieved through highly complex intracellular machineries, encompassing protections of DNA, RNA, proteins, small molecular compounds, organelles, membranes, and their states and structures. Tardigrades are often cross-tolerant to radiation damage even in their active states presumably as a result of their adaptation to desiccating environments, and we therefore searched for the common machinery in anhydrobiosis and UVC tolerance to focus on and to elucidate the tardigrade-unique anti-oxidative stress response mechanisms. Detailed mode of action of specific components may be different between anhydrobiosis and UVC tolerance, but by focusing on the intersection of these stress conditions, we aimed to initially screen for candidate genes through transcriptomic approaches. We have used two time-courses in this experiment; “short” time course to observe the cellular response during the decreased movement, possibly reflecting the immediate cellular repair, and “long” time course for the long-term recovery of cellular state. We observed a dynamic change in the transcriptome between 3–4- and 24–36-h after UVC exposure, presumably corresponding to the regulation of repair pathways required to control the damage induced by the exposure. It would have been ideal to compare the transcriptome profiles with anhydrobiosis recovery, however the only available data lacked biological replicates thus making statistically robust analysis infeasible [14]. Hence, we have used transcriptome sequencing data of slow-dried *R. varieornatus* anhydrobiosis entry [11]. Since *R. varieornatus* is capable of rapid desiccation [36], it typically shows very limited transcriptome induction during anhydrobiosis (54 genes). On the contrary, slow-dried specimens show more regulation (307 genes), which includes several genes related to cellular repair. Studies on the anhydrobiotic midge *Polypedilum vanderplanki* have also observed similar inductions of cellular repair related genes [42]. Together, by comparing the transcriptome response against UVC and desiccation, we hypothesized that high credibility anhydrobiosis genes would be identified.

The functional assays conducted here suggest that g12777 is a manganese-dependent peroxidase, independent of other components of the cell. Previous studies have implicated the relationships between anhydrobiosis and peroxidases [22, 27]. Unlike most Mn peroxidases, this protein does not rely on hemes for the peroxidase function [43, 44]. The presence of a disulfide bond close to the Mn-1 binding site is suggestive of the utilization of the disulfide bond as an electron acceptor, which could categorize this protein as a new class of peroxidase. Constructs lacking the disordered region showed impaired anti-oxidative stress functions. Intrinsically unstructured proteins (IUP) are implied to contribute to cellular tolerance in tardigrades [30, 31] and *Drosophila* [45]. IUPs have been proposed to participate in protein stability [46], therefore we hypothesize this disordered region may contribute to protein stability of g12777 proteins. We have also observed that the g12777 protein utilizes Mn^2+^ ions for peroxidase function, while it has a higher affinity for Zn^2+^ ions. Mn^2+^ itself can carry out peroxidase reactions [47], but the catalytic activity of Mn^2+^ alone was not detected with significance in our experiment, whereas g12777 clearly demonstrated significant peroxidase activity. Furthermore, we speculate that this affinity is related to the localization of this protein mainly in the Golgi apparatus since it has a strictly controlled high manganese concentration [48–51], suggesting the antioxidative stress functionality in the Golgi apparatus seems to be crucial for anhydrobiotic survival. The importance of the Golgi apparatus in stress response and regulation has been studied [52–54], and several studies have identified anhydrobiosis related proteins localizing in the Golgi apparatus [55, 56]. These studies further emphasize the importance of the Golgi apparatus in anhydrobiosis, but its contribution to anhydrobiosis has not been discussed in detail to date. We believe that the results of this work can be a starting point for a more comprehensive study of the intracellular mechanisms of anhydrobiosis.

This protein family is conserved widely in Tardigrada, even in *Batillipes* sp., a non-anhydrobiotic heterotardigrade species. Although this intertidal marine inhabiting *Batillipes* is not capable of anhydrobiosis, it is capable of surviving short periods of high and low levels of salinity and inhabiting brackish regions [57, 58], suggesting tolerance against fluctuations in osmolarity. Such fluctuations would induce physiologies partly mirroring anhydrobiosis. This may imply that this protein may have initially contributed to anti-osmotic stress, which may have been used for desiccation tolerance during terrestrialisation. This would also explain the extensive duplication that occurred around the common ancestor of Eutardigrada, as most eutardigrade species are capable of anhydrobiosis. However, this protein family was not induced before or during the timing where we observed decrease in motility, which may imply that it may not have directly contributed to the immediate damage repair induced by UVC exposure. We note that orthologs in *R. varieornatus* and *H. exemplaris* show expression patterns similar to that of CAHS and SAHS, constant expression in *R. varieornatus* (half of the orthologs have TPM > 100), and high induction in *H. exemplaris* (median 18.7 × induction during anhydrobiosis entry). Together with the peroxidative function, we hypothesize that this protein family is a new anhydrobiosis gene. However, functional analysis in animal specimens have been problematic due to this protein family’s multi-copy nature and the lack of high-efficiency gene knock-down/knock-out methods in tardigrades and would be a subject for further studies.

## Conclusions

In conclusion, our results provide a global image of the transcriptomic response against UVC in an extremotolerant tardigrade. Previous studies have proposed the contribution of highly conserved factors and lineage-specific gene families to anhydrobiosis. We here have identified a novel peroxidase protein family that is conserved only in, but widely within Tardigrada, making it the first tardigrade-specific anhydrobiosis-related gene that is conserved throughout the phylum, in contrast to previously identified tardigrade-specific elements that are conserved in only Eutardigrada at most. While the importance of anti-oxidative stress has been extensively discussed in anhydrobiotic animals, a novel multi-copy peroxidase family this widely conserved may indicate this gene family may be one of the central components of the anhydrobiosis machinery in tardigrades. Thus, these findings underline a fundamental basis of the cellular protection against oxidative stress during anhydrobiosis and the contribution of the Golgi apparatus.

## Materials and methods

### Tardigrade culture

In this study, we used the *Ramazzottius varieornatus* strain YOKOZUNA-1 [36]. The animals were reared in an environment established by our previous study [36] and were maintained in 90 mm petri dish plates. Approximately 600 animals were placed on 2.0% Bacto agar layered dishes and fed with *Chlorella vulgaris* Beijerinck 1890 mixed in Volvic water. The petri dish plates were lidded and placed in a dark incubator, set at 22 ˚C. The animals were transferred to new plates every 7 days.

### Quantification of movement in UVC exposed *R. varieornatus*

Approximately 100 *R. varieornatus* specimens were collected from the culture plate and placed on a single new clean agar plate. After removal of excess water, specimens were exposed to UVC using the UVC lamp of a drying rack (Asone). Removal of excess water does not induce anhydrobiosis and is required to minimize UVC absorbance by the layer of water [6]. The plates were then set for exposure to 2.5 kJ/m^2^ UVC by a UVC lamp. The average dosage per second was 0.54 mW/cm^2^s (0.0054 kJ/m^2^s) at room temperature, quantified by UV intensity meter (Fuso, YK-37UVSD), thus the exposure took 463.0 s. Water was added to the plates immediately after irradiation and ten individuals were randomly picked and placed into a single well of the 6 × 12 plate layered with 2 µL of 2% agar gel (UVC exposed sample). This UVC exposure was repeated for five times (5 biological replicates). Additionally, 50 individuals were collected from the culture and ten individuals each were placed into a single well (control samples, 5 biological replicates). The tardigrade specimens were set to incubate in a dark chamber set at 22˚C. Each well with tardigrade specimens were supplied after the first time point (T1) with *C. vulgaris* to prevent starvation. A 30 s movie was taken at 0, 1, 2, 3, 6, 9, 12, 18, 24, 36, 48, and 72 h after exposure for each individual with VHX-5000 (Keyence). An individual was classified as moving if any movement was observed within this thirty-second time frame. The quantified movement was statistically compared with a non-irradiated control group by ANOVA and Tukey HSD in R. Conditions with FDR < 0.05 were classified as significant changes.

### Transcriptome sequencing of UVC exposed *R. varieornatus* specimens

We conducted RNA-Seq on two time-courses with three biological replicates, the first 0–12 h post exposure (1, 2, 3, 4, 5, 6, 9 and 12 h), designated “Short”, and the second 0–72 h post exposure (12, 24, 36, 48 and 72 h), designated “Long”. For UVC exposure, 300 and 210 individuals were collected from culture plates (4 biological replicates) and was exposed to 452 (0.554 mW/cm^2^) and 438 s (0.57 mW/cm^2^) UVC for Short and Long time courses, respectively. Water was added immediately after UVC exposure and specimens were incubated at 22˚C until sampling. For the Long time course, the agar plates were supplied with *C. vulgaris* at the 12-h time point to prevent starvation. With each sampling, randomly chosen 30 individuals were placed in a PCR tube with minimal water and preserved at -80˚C. For controls, randomly chosen 30 individuals were collected as above without UVC exposure at time point 0 h and placed into a PCR tube (4 biological replicates).

For RNA-Seq library construction, frozen samples were first immersed in 100 µL of TRIzol reagent (ThermoFisher Scientific) and homogenized with Biomasher II (Nippi. Inc.). Additional 200 µL of TRIzol reagent was added for total RNA extraction with Direct-zol (Zymo Research). Three samples were chosen for each condition by RIN values calculated by electrophoresis by TapeStation High Sensitivity RNA tape (Agilent Technologies). Total RNA was submitted to mRNA Isolation and RNA fragmentation with NEBNext Oligo d(T)25 beads (New England BioLabs) and cDNA synthesis, adapter ligation and PCR enrichment with NEBNext Ultra RNA Library Prep Kit (New England BioLabs). RNA and cDNA concentrations were determined using Qubit RNA/DNA (ThermoFisher Scientific), and electrophoresis of the synthesized library was conducted with TapeStation D1000 tape (Agilent Technologies). After pooling all of the samples (70 ng of cDNA per sample), the library was size-selected for fragments between 300–1,000 bp with an E-Gel EX 1% (ThermoFisher Scientific) and NucleoSpin Gel and PCR Clean-up (Takara). The sequencing library was sequenced with the Illumina Next-Seq 500 instrument (Illumina) with 75 bp single end settings.

### Informatics analysis

Prior to informatics analysis, preparation of sequenced reads was conducted. After de-multiplexing sequenced raw files with bcl2fastq (Illumina), quality was checked with FastQC [59]. We then used Kallisto [60] (v0.44.0, –boot 1000, –bias) to quantify gene expression, using the *R. varieornatus* genome [11, 14]. To determine differentially expressed genes (DEGs), we mapped the RNA-Seq reads to the coding sequences with BWA MEM (v0.7.12-r1039) and conducted statistical test with DESeq2 (v1.22.2) [61, 62]. Genes with FDR below 0.05 and fold change over 2 were determined as DEGs. The expression profiles were validated using Spearman correlation in between samples and clustering. DEGs of slow-dried *R. varieornatus* anhydrobiotic samples were obtained from our previous study [11], and differentially expressed genes in both conditions were obtained. The conservation of commonly differentially expressed genes was submitted to BLASTP (v2.2.22) search against the predicted proteome sequences indicated below. Gene ontology enrichment analysis was conducted with in-house scripts based on the Gene Ontology terms associated with Swiss-Prot homologs or with GOStats based on InterProScan annotations [63].

For the identification of OG0000230 and OG0000231 orthologs, we first prepared published transcriptomes and our in-house tardigrade genome database (Arakawa, Personal communication). We obtained transcriptome assemblies of *Richtersius coronifer* Richters 1903 and *Echiniscoides sigismundi* Schultze 1865 from previous studies [15, 64, 65]. Additionally, we re-assembled each transcriptome using Bridger [66] (v2014-12–01, *R. coronifer*: SRR7340056, *E. sigismundi*: SRR7309271, default parameters). Each tardigrade genome/transcriptome was submitted to BUSCO genome completeness validation against the eukaryote lineage [67]. The gene model obtained during this assessment was used with autoAugPred.pl script in the Augustus (v3.3.3) package to predict genes in each genome [68]. The amino acid sequences of each genome were submitted to a TBLASTX or BLASTP search (v2.2.22) [69] of g12777.t1 and g241.t1 coding sequences and amino acid sequences to obtain orthologs in each species. Additionally, the g241 amino acid sequence was submitted to a Diamond BLASTP (v0.9.24.125) search against NCBI NR and NCBI Bacteria RefSeq to obtain non-tardigrade orthologs [70, 71]. Furthermore, we submitted predicted proteome sequences for each tardigrade species and additional metazoan species used in our previous study [11] to OrthoFinder (v2.3.7, –mcl-I 2.0) to validate our BLASTP search results [72]. For OG0000231 orthologs, amino acid sequences were subjected to MAFFT (v7.271, –auto) multiple sequence alignment and phylogenetic tree construction by FastTree (v2.1.8 SSE3, -b 1000, -gamma) [73, 74], which was visualized in iTOL [75]. Orthologs were also submitted to MEME analysis (v5.1.0) for a novel motif search [76]. Disordered regions in the g12777 protein were predicted with DISOPRED and IUPRED [77, 78]. G-language Genome Analysis Environment (v1.9.1) was used for data manipulation [79, 80]. Furthermore, *R. varieornatus* and *H. exemplaris* OG0000231 orthologs were submitted to constraint-based multiple alignment tool (COBALT v1.19.1) for visualization (https://www.ncbi.nlm.nih.gov/tools/cobalt/cobalt.cgi).

### Sample preparation of recombinant proteins

For biochemical and biophysical characterizations of the g12777 protein with deletions of the N-terminal signal sequence and putative intrinsically disordered region, the gene encoding residues Gly63–Leu231 were cloned into the *Nde*I and *Eco*RI sites of the pET-28b vector (Novagen—Merck Millipore). *Escherichia coli* BL21-CodonPlus (DE3) harboring the plasmids were cultured in LB medium containing 15 µg/ml kanamycin and subsequently harvested after induction with 0.5 mM isopropyl β-D-thiogalactoside for 4 h at 37 °C. The harvested cells were resuspended with buffer A [20 mM Tris–HCl (pH 8.0), 150 mM NaCl, and 1 mM EDTA] and lysed by sonication. The insoluble inclusion bodies were extensively washed with buffer A in the presence and absence of 2% Triton X-100, and then solubilized with 6 M guanidinium chloride, 50 mM Tris–HCl (pH 8.0), and 0.5 mM dithiothreitol. The solubilized proteins were refolded by dilution (0.05 mg/mL) in 50 mM Tris–HCl (pH 8.0), 400 mM L-arginine, 5 mM reduced glutathione, 0.5 mM oxidized glutathione, and 2 mM CaCl_2_ at 4 °C for 12 h. The refolded protein was concentrated and then dialyzed against 20 mM Tris–HCl (pH 8.0), 150 mM NaCl, and 2 mM CaCl_2_. Subsequently, the N-terminal His6-tag peptide was removed by thrombin digestion. The nontagged proteins were incubated at 4 °C for 30 min in the presence of 10 mM EDTA and 0.1 mM AEBSF [4-(2-Aminoethyl)benzenesulfonyl fluoride hydrochloride], and further purified with a HiLoad Superdex 75 pg (GE Healthcare) equilibrated with buffer A.

### Crystallization, X-ray data collection and structure determination

The catalytic domain of g12777 protein (10 mg/mL) was dissolved in 20 mM Tris–HCl (pH 8.0), 150 mM NaCl and 2 mM CaCl_2_. The crystals of Zn^2+^-bound g12777 protein complex were obtained in a precipitant containing 10% PEG3350, 100 mM imidazole–HCl buffer (pH 7.5), 300 mM zinc acetate, and 50 mM sodium fluoride upon incubation at 20 °C for 3 days. In contrast, the crystals of Mn^2+^-bound complex were obtained by a soaking with a buffer containing 11% PEG3350, 100 mM imidazole–HCl buffer (pH 7.5), 50 mM manganese chloride, and 50 mM sodium fluoride for 5 h using the Zn^2+^-bound crystals. The crystals were cryoprotected with the crystallization or soaking buffer supplemented with 20% glycerol. The crystals of Zn^2+^- and Mn^2+^-bound g12777 protein complexes belonged to space groups *P*1 and *P*2_1_ with two g12777 protein molecules per asymmetric unit and diffracted up to resolutions of 1.60 Å and 2.30 Å, respectively. Diffraction data were scaled and integrated using XDS (GPLv2) [81] and AIMLESS (ver. 1.12.1) [82].

The crystal structure of the catalytic domain of the g12777 protein crystallized in the presence of excess amount of Zn^2+^ ion (300 mM) was solved by the single-wavelength anomalous dispersion (SAD) method using 1.1200 Å wavelength (Aichi-SR BL2S1) with the program Autosol in the Phenix suite (ver. 1.11.1–2575) [83]. As for the Mn^2+^-bound g12777 protein complex, the crystal structure was solved by the molecular replacement method using the Zn^2+^-bound structure as a search model. The diffraction data set was collected at Osaka University using BL44XU beamline at SPring-8 (Japan). Automated model building and manual model fitting to the electron density maps were performed using ARP/wARP (ver. 8.0) [84] and COOT (ver. 0.9) [85], respectively. REFMAC5 (ver. 5.8.0258) [86] was used to refine the crystal structure, and the stereochemical quality of the final models were validated using MolProbity (ver. 4.2) [87], showing that no amino acids were located in the disallowed regions of the Ramachandran plot. The final model of the Zn^2+^-bound g12777 protein complexes had *R*_work_ of 19.2 and *R*_free_ of 24.5%, whereas that of Mn.^2+^-bound form had *R*_work_ of 26.2 and *R*_free_ of 32.5% (Supplementary Table S2). The molecular graphics were prepared using PyMOL (ver. 2.4.0a0, https://pymol.org/2/). These structures were compared with known protein structures using the DALI server [88]

### Measurement of enzymatic activity of the g12777 protein

The catalase/peroxidase activities of the g12777 protein were measured using Cayman Catalase Assay Kit (Cayman Chemical) following the manufacturer’s protocol. This assay is based on the peroxidatic reaction of H_2_O_2_ + CH_3_OH → HCHO + 2H_2_O, measured colorimetrically by employing 4-amino-3-hydrazino- 5-mercapto-1,2,4-triazole (Purpald) as the chromogen. For the enzymatic assay, 20 ng of the wild type g12777 protein catalytic domain was incubated in the presence and absence of 2 mM ZnCl_2_ and MnCl_2_ for 30 min at 25 °C. Bovine liver catalase (Sigma Aldrich) was used as a positive control. Catalase/peroxidase activity were tested with ANOVA and Tukey HSD in R.

### Isothermal titration calorimetry

Purified catalytic domain of the g12777 protein dissolved in 20 mM Bis–Tris-HCl (pH 6.5) containing 150 mM NaCl was used for isothermal titration calorimetry (ITC). In this experiment, a syringe containing 1 mM ZnCl_2_, MnCl_2_ or CaCl_2_ was titrated into a sample cell containing 0.1 mM catalytic domain of the g12777 protein using an iTC200 calorimeter (GE Healthcare).

### Expression of GFP-tagged g12777 in HEK293

For expression of GFP-fused g12777 proteins, we inserted the full coding sequence between the *Sal*I and *BamH*I restriction sites of the pAcGFP1-N1 plasmid (Takara). The total RNA was extracted from *R. varieornatus* with Direct-zol Ultra RNA (Zymo Research) and was reverse transcribed with PrimeScript Reverse Transcriptase (Takara). g12777 mRNA was selected by PCR using Tks Gflex DNA Polymerase (Takara) with the following primers:

- g12777-F: 5’-ATTGTCGACATGGCATTATCTTTGTGGATGACTG-3’,
- g12777-R: 5’-TAAGGATCCTTTAGGGAAGAAGGTGCCCGACAG-3’.

For construction of Δ62aa construct, the forward primer was changed to 5’-ATTGTCGACGGCCGCTTTGCCGATTTCTTCAGAA-3’. The corresponding g12777-D2A (D92A, D98A, D161A, D163A mutations, g12777-D2A) coding sequences were adapted for human codon usage and synthesized at Eurofin Genomics. g12777-D2A was inserted into the pAcGFP1-N1 plasmid (Takara) between and *Sal*I-*BamH*I. Constructs were transfected into HEK293 cells with the X-tremeGENE 9 reagent (Sigma-Aldrich) and submitted to selection with G418 (Sigma-Aldrich) at 400 µg/mL for more than two weeks, and further passaged at 100 µg/mL. HEK293 cell lines were cultured with MEM medium (Sigma-Aldrich) supplied with FBS (Funakoshi), NEAA (ThermoFisher Scientific) and Antibiotic–Antimycotic Mixed Stock Solution (Nacalai Tesque) and were passaged every 3–4 days with TryPLE (ThermoFisher Scientific). For microscopy observations, cells were transformed with 20 µL of CellLight Golgi RFP, BacMan 2.0 (ThermoFisher Scientific) and then fixed with 4% Paraformaldehyde in PBS (Wako) and stained with 300 nM DAPI (ThermoFisher Scientific) to visualize the nucleus and Golgi apparatus, respectively. Fluorescent signals were observed with and all-in-one fluorescence microscope SZ-5000 (KEYENCE) equipped with an advanced observation module (BZ-H3XD, KEYENCE), optical sectioning module (BZ-H3XF, KEYENCE), CFI Plan Apo λ 40 × objective (972,033, NA 0.95, KEYENCE) or CFI Plan Apo λ 100 × H objective (972,037, NA 1.45, KEYENCE) and sectioned images were analyzed with the BZ-X Analyzer software (BZ-H3A, KEYENCE). DAPI, GFP, and TRITC filters were used (OP-87762, OP-87763, OP-87764, KEYENCE).

Additionally, the localization of six high expressed OG0000231 orthologs were validated using similar methods. cDNA was obtained from *R. varieornatus* with RNeasy Mini Kit (Qiagen) and PrimeScript II 1st strand cDNA Synthesis Kit (Takara). The coding sequences of the corresponding genes were amplified by PrimeSTAR Max DNA Polymerase (Takara) using the following primers:

- g243-F: 5’-ATTCTGCAGTCGACGGTATGCAGGATGTTTCCGA-3’,
- g243-R: 5’-catgaccggtggatcCTATTTGGTGAACCATCCG-3’,
- g244-F: 5’-ATTCTGCAGTCGACGATGGCCAAGGCGGCAATC-3’,
- g244-R: 5’-catgaccggtggatcTCATCTAGAGAAAAGTCCGC-3’,
- g245-F: 5’-ATTCTGCAGTCGACGGTATGAACTTTCTCTGCTGG-3’,
- g245-R: 5’-catgaccggtggatcTCAGGAGAACATGCCCGA-3’,
- g246-F: 5’-ATTCTGCAGTCGACGATGATGCAGCTGACAATCTT-3’,
- g246-R: 5’-catgaccggtggatcTTATGAGAACATGCCGTCG-3’,
- g779-F: 5’-ATTCTGCAGTCGACGGTATGGATCTGGACAGGG-3’,
- g779-R: 5’-catgaccggtggatcTCATTTTCCAATAAAGGGAATG-3’,
- g2856-F: 5’-ATTCTGCAGTCGACGGTATGTGGGGAATACTGTG-3’,
- g2856-R: 5’-catgaccggtggatcTCATGCCATAGCGTGGCG-3’,
- g12777-F: 5’-ATTCTGCAGTCGACGGTATGGCATTATCTTTGTGG-3’,
- g12777-R: 5’-catgaccggtggatcTTATAGGGAAGAAGGTGCC-3’,

These coding sequences were inserted into the pAcGFP1-N1 plasmid by In-Fusion HD Cloning Kit. Constructs were transfected into HEK293 cells with the X-tremeGENE 9 reagent (Sigma-Aldrich) in optiMEM solution for 4 h. After incubation for 24–48 h in MEM medium, cells were co-stained with Hoechst33342 for DNA staining (ThermoFisher Scientific) and CellLight Golgi-RFP (ThermoFisher Scientific). Fluorescent signals were observed with a confocal laser scanning microscope TCS SP8 (Leica Microsystems) equipped with HC PL APO CS2 100 × H objective (11,506,372, NA 1.4, Leica Microsystems). Hoechst33342, mEGFP and CellLight Golgi-RFP signals were detected with sequential scanning on Hybrid detector (HyD), laser wavelength and filter setting were selected to 405, 474, and 551 nm, and 409–476, 484–554, and 554–622 nm, respectively. Confocal images were analyzed with ImageJ.

### Validation of cellular tolerance against oxidative stress

To evaluate cellular tolerance of g12777 transfected cells, we conducted MTT analysis of H_2_O_2_ exposed cell lines expressing only GFP, g12777, g12777-Δ62aa, and g12777-D2A (3 technical replicates). In brief, approximately 10,000 cells were plated into 96 well plates and were incubated at 37˚C 5% CO_2_ for 24 h. Cells were exposed to 0–1.0 mM H_2_O_2_ (Wako) for 30 min (3–5 replicates). The culture medium was removed to stop exposure, and 100 µL MEM medium was added to the wells and further incubated for 24 h. The culture medium was replaced with 100 µL fresh culture medium supplied with 10 µL of 5 µg/µL Thiazolyl Blue Tetrazolium Bromide (Sigma-Aldrich) and incubated for 2 h for formazan formation. The culture medium was removed, and 100 µL DMSO (Wako) was added to melt the formazan. The 96 well plate was measured with 570 nm and 670 nm using SpectraMax Plus 384 (Molecular Devices). Three technical replicates were measured. Absorbance values were calculated by Ab570 – Ab670 – Ab-BLANK. Absorbance values were statistically tested with ANOVA and Tukey HSD in the R program.

For MACS flow cytometry, cells were cultured and exposed to H_2_O_2_ similar to the MTT assay protocol. After 24-h incubation after exposure, the MEM medium (100 µL) was moved to a clean well and each well was washed with 100 µL PBS (-). All of the PBS was moved to the corresponding well. 50 µL TryPLE was added for trypsinization and incubated at 37˚C in the CO_2_ incubator for 4 min. The MEM culture and PBS mixture were moved to their original wells and mixed thoroughly to release the cells. The 96 well plate was centrifuged at 2,000 rpm (750 g) for 6 min at 4˚C, and the supernatant was removed. Each well was washed with 1 × Annexin binding buffer (ThermoFisher Scientific), and was centrifuged at 2,000 rpm (750 g) for 6 min at 4˚C. The buffer was removed, and each well was supplied with 100 µL of Annexin binding buffer supplied with 0.1 µL SYTOX™ Blue Nucleic Acid Stain (ThermoFisher Scientific) and 2 µL Annexin V Alexa Fluor 657 (ThermoFisher Scientific). The cells were resuspended with pipetting and set to stain for 10 min on ice. The MACS Quant10 instrument (Miltenyi Biotec) was set for measurement of DAPI, Alexa 657 and GFP. Three technical replicates were measured. Approximately 100–1,500 cells were measured for each cell line. The ratio of healthy cells (Anninx-V (-) SYTOX blue (-) cells) were compared with ANOVA and Tukey HSD in the R program. Adjusted *p*-values (FDR) below 0.05 were designated as significant differences.

## Supplementary Information

**Additional file 1:** **Figure S1. **Expression profiles of UVC exposed *R. varieornatus*. Gene expression profiles of *R. varieornatus *specimens exposed to 2.5kJ/m^2^ UVC shown as a heatmap. TPM values were Z-scaled to between samples. Expression and sample profiles were clustered with by Ward method based on Spearman correlation and the cluster number is indicated on the right side of the heatmap (Cluster numbers from one to eight). Samples are indicated in the following format: Short or long, time point, replicate number. **Figure S2. **Phylogenetic analysis OG0000230 orthologs within Tardigrada. Phylogenetic tree of tardigrade OG0000230 orthologs and bacterial orthologs. Amino acid sequences were aligned with Mafft, and the phylogenetic tree was constructed with FastTree with 1000 bootstraps (-boot, -gamma). CAHS genes were used as an out-group. **Figure S3. **Multiple alignment of *R. varieornatus* and *H. exemplaris* OG0000231 orthologs. The amino acid sequences of OG0000231 orthologs were submitted to multiple alignment by constraint-based multiple alignment tool from NCBI. **Figure S4. **Metal-binding property and structural detail of g12777 globular domain. [a,b] The crystal structures of catalytic domain of g12777 complexed with Mn^2+^ [a] andZn^2+^ [b]. All crystallographically observed metal ion binding sites are indicated. [c] Topology diagram of g12777 catalytic domain. [d] Close-up view of the Zn^2+^-binding site. The residues comprising binding site 1(D92, D98, D161, and D163) and the disulfide bond (C91 and C97) are indicated. [e] Conservation of residues. All OG0000231 orthologs were submitted to MEME search. [f] Conserved resides of g12777. The conserved resides are colored according to the bit scores obtained from MEME analysis. [g] Ca^2+^and Zn^2+^ ion binding affinity measured by isothermal titration calorimetry. The upper two panels indicate the raw data, while the bottom two panel represent the integrated heat values corrected for the heat of dilution and fit to a one-site binding model (solid line); red: Ca^2+^, cyan: Zn^2+^. Time is in seconds. **Figure S5. **Localization of other OG0000231 orthologs. The localization of top six highly expressed OG0000231 orthologs were validated using the same method of Figure 3a. All of these orthologs showed localization mainly to the Golgi apparatus. The images have been adjusted for visualization. **Figure S6. **Comparison of peroxidase function with Bovine liver catalase. The peroxidase function of g12777 + Mn^2+^ was compared to those of Bovine liver catalase (BLC). *p*-value < 0.001 (Welch’s t-test).

**Additional file 2:** **Table S1. **Statistics of RNA-Seq data obtained in this study. Statistics of each RNA-Seq data sequenced in this study. Mapping ratio for BWA mapping are shown. **Table S2. **Highly expressed or regulated genes in Short time-course. Genes with TPM values > 1,000 and fold change > 4 are highlighted in purple and orange, respectively. **Table S3. **Highly expressed or regulated genes in Long time-course. Genes with TPM values > 1,000 and fold change > 4 are highlighted in purple and orange, respectively. **Table S4. **Annotation of conserved genes. Details of the genes with Swiss-Prot homologs. **Table S5. **Annotation of novel genes. Details of the genes without Swiss-Prot homologs. Conservation profiles within Tardigrada based on OrthoFinder results, domains identified by InterProScan and the amino acid sequences are indicated. **Table S6. **OG0000231 and OG0000230 orthologs within Tardigrada identified by BLASTP. Coding sequences of OG0000230 and OG0000231 proteins were submitted to BLASTP search against predicted proteome sequences of indicated species in our in-house genome database and additional transcriptome assemblies. **Table S7. **OG0000231 copy numbers within Tardigrada identified by Orthofinder. Number of genes classified as OG0000231 orthologs by Orthofinder. **Table S8. **OG0000230 copy numbers within Tardigrada identified by Orthofinder. Number of genes classified as OG0000230 orthologs by Orthofinder. **Table S9. **Expression of OG0000230 orthologs. TPM values of OG0000230 orthologs in *R. varieornatus* UVC response and anhydrobiosis. **Table S10. **Expression of OG0000231 orthologs. TPM values of OG0000231 orthologs in *R. varieornatus*UVC response and anhydrobiosis. **Table S11. **Refinement of OG0000231 gene regions. Mispredicted OG0000231 orthologs in *R. varieornatus* genome. **Table S12. **HGT statistics of OG0000230 orthologs from previous studies. HGT statistics calculated in our previous study [11] for OG0000230 orthologs. **Table S13. **OG0000230 orthologs identified from NCBI nr database. List of orthologs identified by Diamond BLASTP search (E-value < 1E-5) against NCBI nr database. Tardigrade, non-tardigrade eukaryotic, and bacterial hits were each colored in gray, yellow, and green. *H. exemplaris* orthologs colored in red had gene annotations, but validation by RNA-Seq data mapping indicated that these genes may be mis-predicted. **Table S14. **OG0000230 orthologs identified from RefSeq database. List of orthologs identified by Diamond BLASTP search (E-value < 1E-5) against NCBI Bacteria RefSeq complete genome sequences. Genes before and after OG000023 orthologs are shown. **Table S15. **Gene synteny of OG0000231 and OG0000230 orthologs in *R. varieornatus. *Annotation of five genes prior and after OG0000231 orthologs in* R. varieornatus.* OG0000231 and OG0000230 orthologs are highlighted in red. **Table S16. **Data collection and refinement statistics for g12777. Crystal parameters and refinement statistics for Mn^2+^- and Zn^2+^-bound g12777 protein catalytic domain are summarized. One crystal for each structure was used for diffraction data collection. **Table S17. **Metal ion affinity of recombinant proteins. Affinity values for metal ions with g12777 proteins.

## Data Availability

The coordinates and structural factors of the crystal structure of the catalytic domain of g12777 protein complexed with Mn^2+^ or Zn^2+^ ions have been deposited in the Protein Data Bank under accession numbers 7DBT and 7DBU, respectively. The raw X-Ray diffraction data used to produce protein structures have been uploaded to XRDa under the DOI https://doi.org/10.51093/xrd-00078 and https://doi.org/10.51093/xrd-00079. The RNA-Seq data obtained were deposited to NCBI GEO under the accession ID GSE152753. The transcriptome assemblies of *Richtersius coronifer* Richters 1903 and *Echiniscoides sigismundi* Schultze 1865 from https://doi.org/10.17894/ucph.9d3a898c-37bb-4cd1-909f-e7fcb07e58d9 and their corresponding transcriptome sequencing datasets from NCBI SRA (*R. coronifer*: SRR7340056, *E. sigismundi*: SRR7309271). The RefSeq and NCBI NR entries were downloaded from NCBI FTP (ftp://ftp.ncbi.nlm.nih.gov).

## Abbreviations

FDR: False discovery rate
ROS: Reactive oxygen species
TPM: Transcript per million
UVB: Ultraviolet B
UVC: Ultraviolet C
