# Supplementary figures and images for: Time-series transcriptomic screening of factors contributing to the cross-tolerance to UV radiation and anhydrobiosis in tardigrades
Source: BMC Genomics. 2022 May 28;23:405. doi: 10.1186/s12864-022-08642-1 (PMC9145152; doi:10.1186/s12864-022-08642-1)

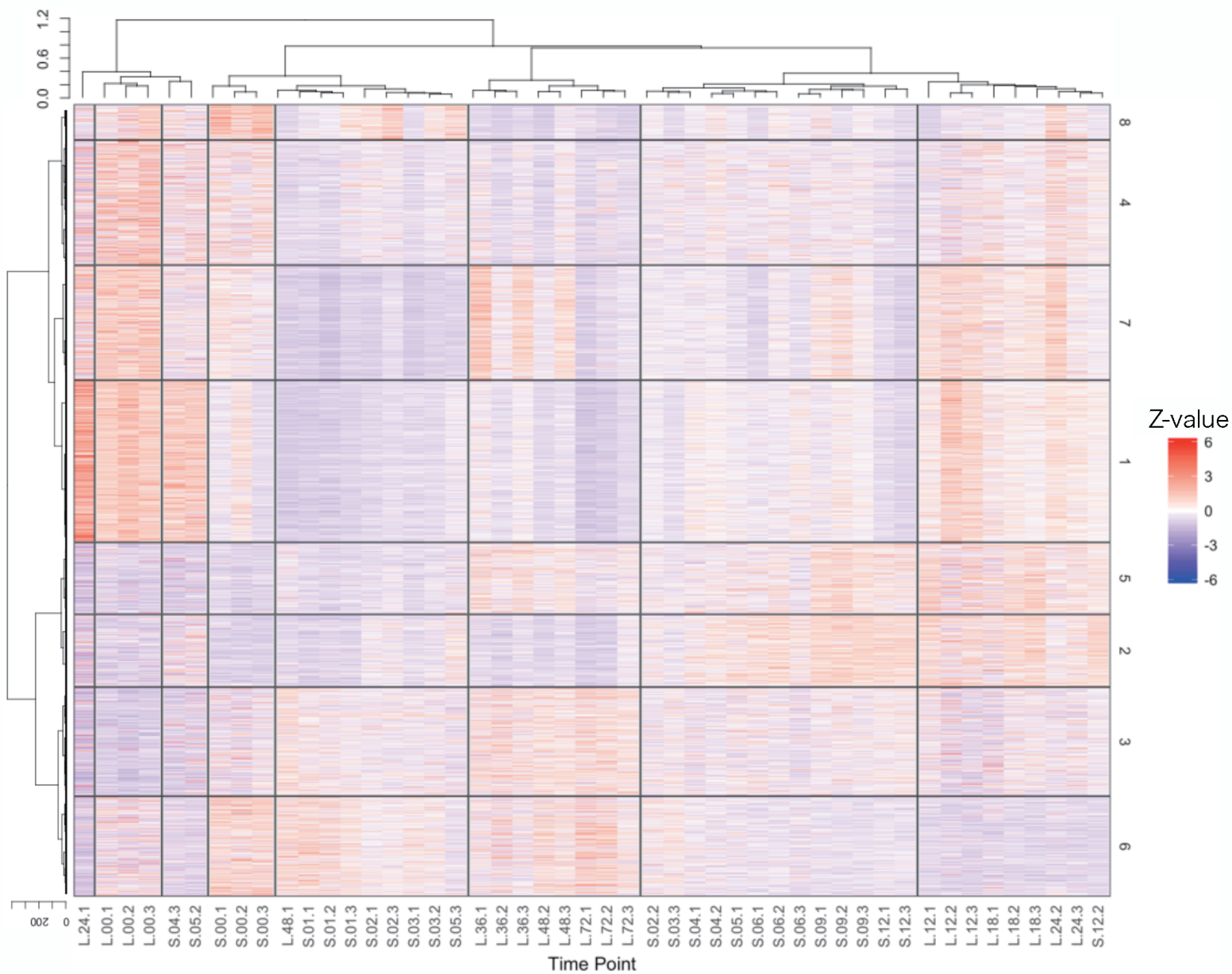

Supplement: Supplementary file 1 — Additional file 1: Figure S1. Expression profiles of UVC exposed R. varieornatus. Gene expression profiles of R. varieornatus specimens exposed to 2.5kJ/m2 UVC shown as a heatmap. TPM values were Z-scaled to between samples. Expression and sample profiles were clustered with by Ward method based on Spearman correlation and the cluster number is indicated on the right side of the heatmap (Cluster numbers from one to eight). Samples are indicated in the following format: Short or long, time point, replicate number. Figure S2. Phylogenetic analysis OG0000230 orthologs within Tardigrada. Phylogenetic tree of tardigrade OG0000230 orthologs and bacterial orthologs. Amino acid sequences were aligned with Mafft, and the phylogenetic tree was constructed with FastTree with 1000 bootstraps (-boot, -gamma). CAHS genes were used as an out-group. Figure S3. Multiple alignment of R. varieornatus and H. exemplaris OG0000231 orthologs. The amino acid sequences of OG0000231 orthologs were submitted to multiple alignment by constraint-based multiple alignment tool from NCBI. Figure S4. Metal-binding property and structural detail of g12777 globular domain. [a,b] The crystal structures of catalytic domain of g12777 complexed with Mn2+ [a] andZn2+ [b]. All crystallographically observed metal ion binding sites are indicated. [c] Topology diagram of g12777 catalytic domain. [d] Close-up view of the Zn2+-binding site. The residues comprising binding site 1(D92, D98, D161, and D163) and the disulfide bond (C91 and C97) are indicated. [e] Conservation of residues. All OG0000231 orthologs were submitted to MEME search. [f] Conserved resides of g12777. The conserved resides are colored according to the bit scores obtained from MEME analysis. [g] Ca2+and Zn2+ ion binding affinity measured by isothermal titration calorimetry. The upper two panels indicate the raw data, while the bottom two panel represent the integrated heat values corrected for the heat of dilution and fit to a one-site bindin [file 12864_2022_8642_MOESM1_ESM.zip › fig_S1.pdf]

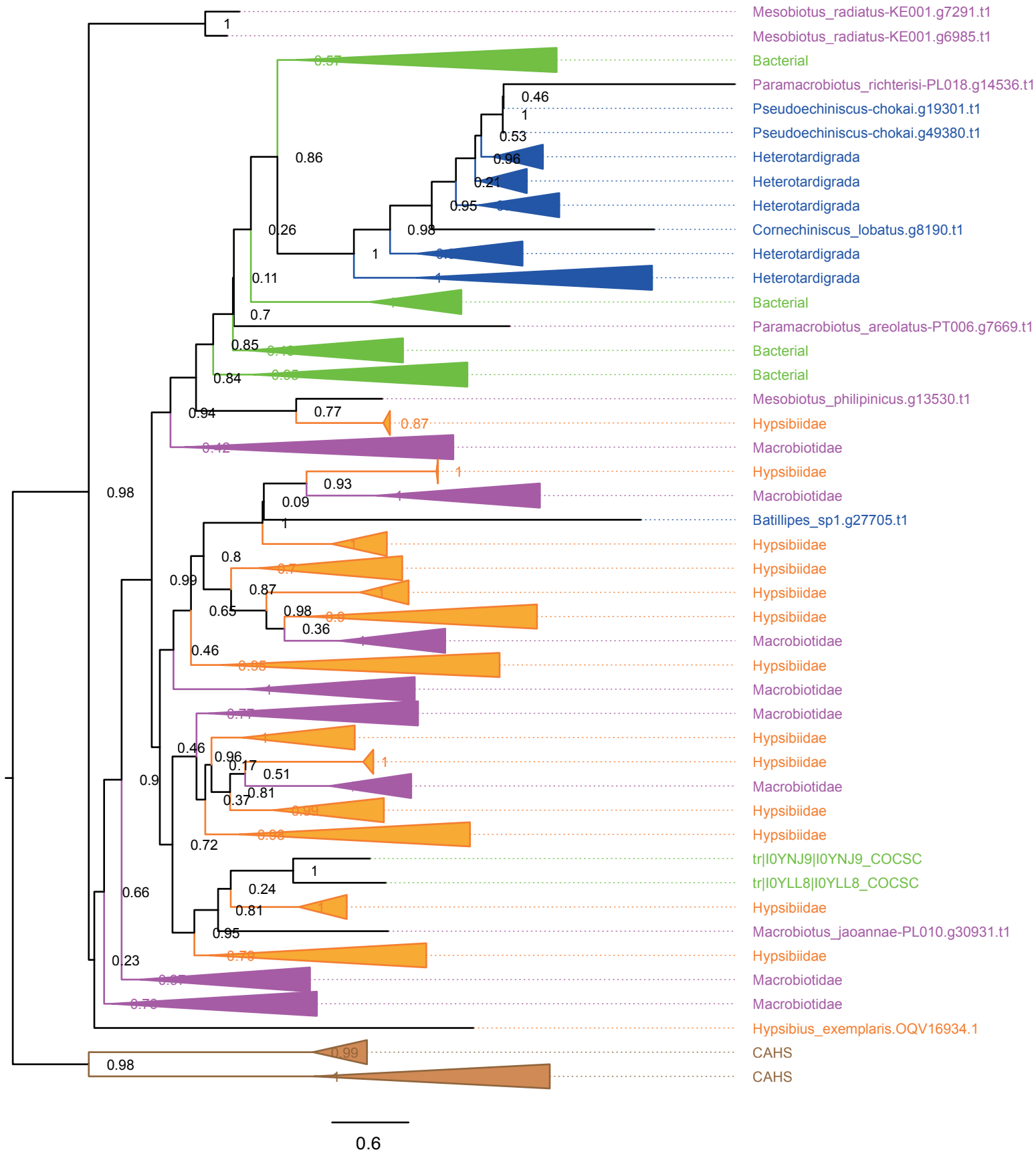

Supplement: Supplementary file 1 — Additional file 1: Figure S1. Expression profiles of UVC exposed R. varieornatus. Gene expression profiles of R. varieornatus specimens exposed to 2.5kJ/m2 UVC shown as a heatmap. TPM values were Z-scaled to between samples. Expression and sample profiles were clustered with by Ward method based on Spearman correlation and the cluster number is indicated on the right side of the heatmap (Cluster numbers from one to eight). Samples are indicated in the following format: Short or long, time point, replicate number. Figure S2. Phylogenetic analysis OG0000230 orthologs within Tardigrada. Phylogenetic tree of tardigrade OG0000230 orthologs and bacterial orthologs. Amino acid sequences were aligned with Mafft, and the phylogenetic tree was constructed with FastTree with 1000 bootstraps (-boot, -gamma). CAHS genes were used as an out-group. Figure S3. Multiple alignment of R. varieornatus and H. exemplaris OG0000231 orthologs. The amino acid sequences of OG0000231 orthologs were submitted to multiple alignment by constraint-based multiple alignment tool from NCBI. Figure S4. Metal-binding property and structural detail of g12777 globular domain. [a,b] The crystal structures of catalytic domain of g12777 complexed with Mn2+ [a] andZn2+ [b]. All crystallographically observed metal ion binding sites are indicated. [c] Topology diagram of g12777 catalytic domain. [d] Close-up view of the Zn2+-binding site. The residues comprising binding site 1(D92, D98, D161, and D163) and the disulfide bond (C91 and C97) are indicated. [e] Conservation of residues. All OG0000231 orthologs were submitted to MEME search. [f] Conserved resides of g12777. The conserved resides are colored according to the bit scores obtained from MEME analysis. [g] Ca2+and Zn2+ ion binding affinity measured by isothermal titration calorimetry. The upper two panels indicate the raw data, while the bottom two panel represent the integrated heat values corrected for the heat of dilution and fit to a one-site bindin [file 12864_2022_8642_MOESM1_ESM.zip › fig_S2.pdf]

R. varieornatus

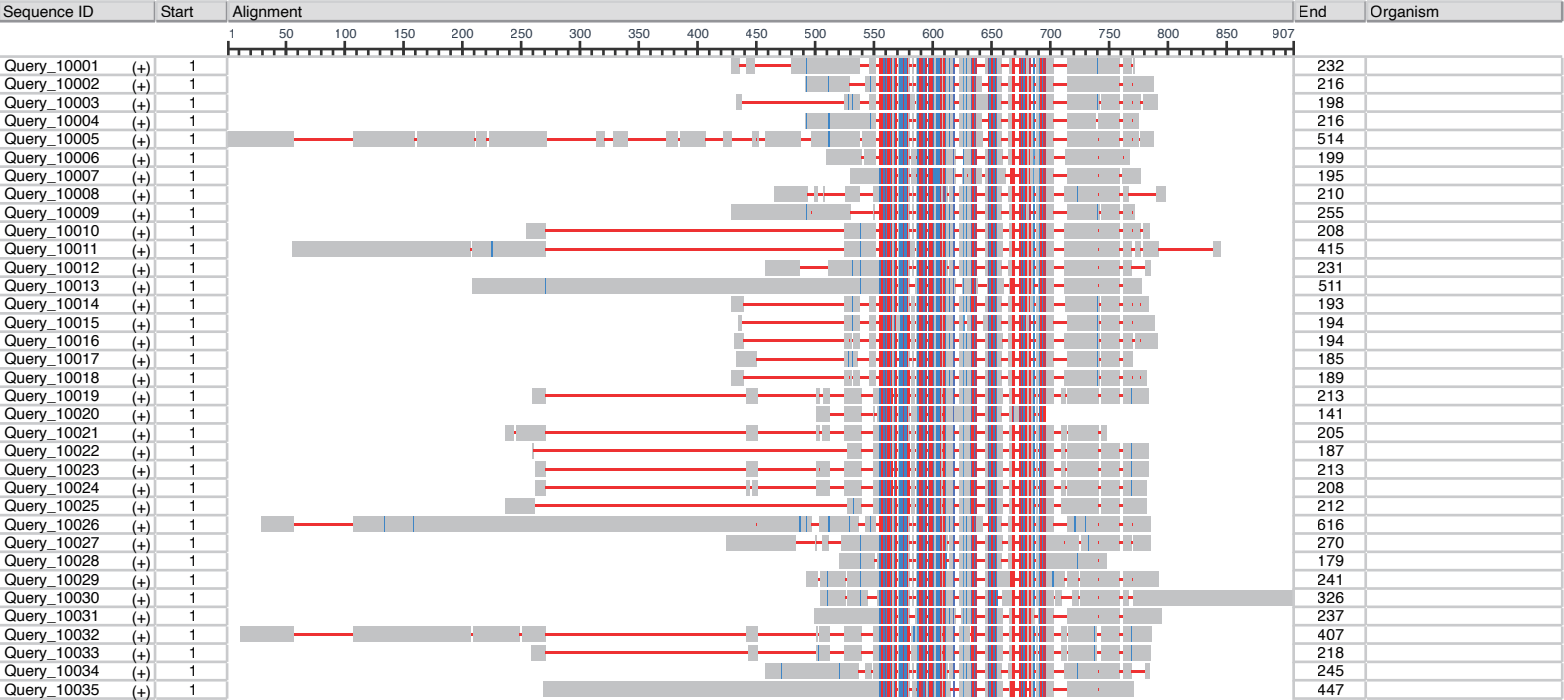

H. exemplaris

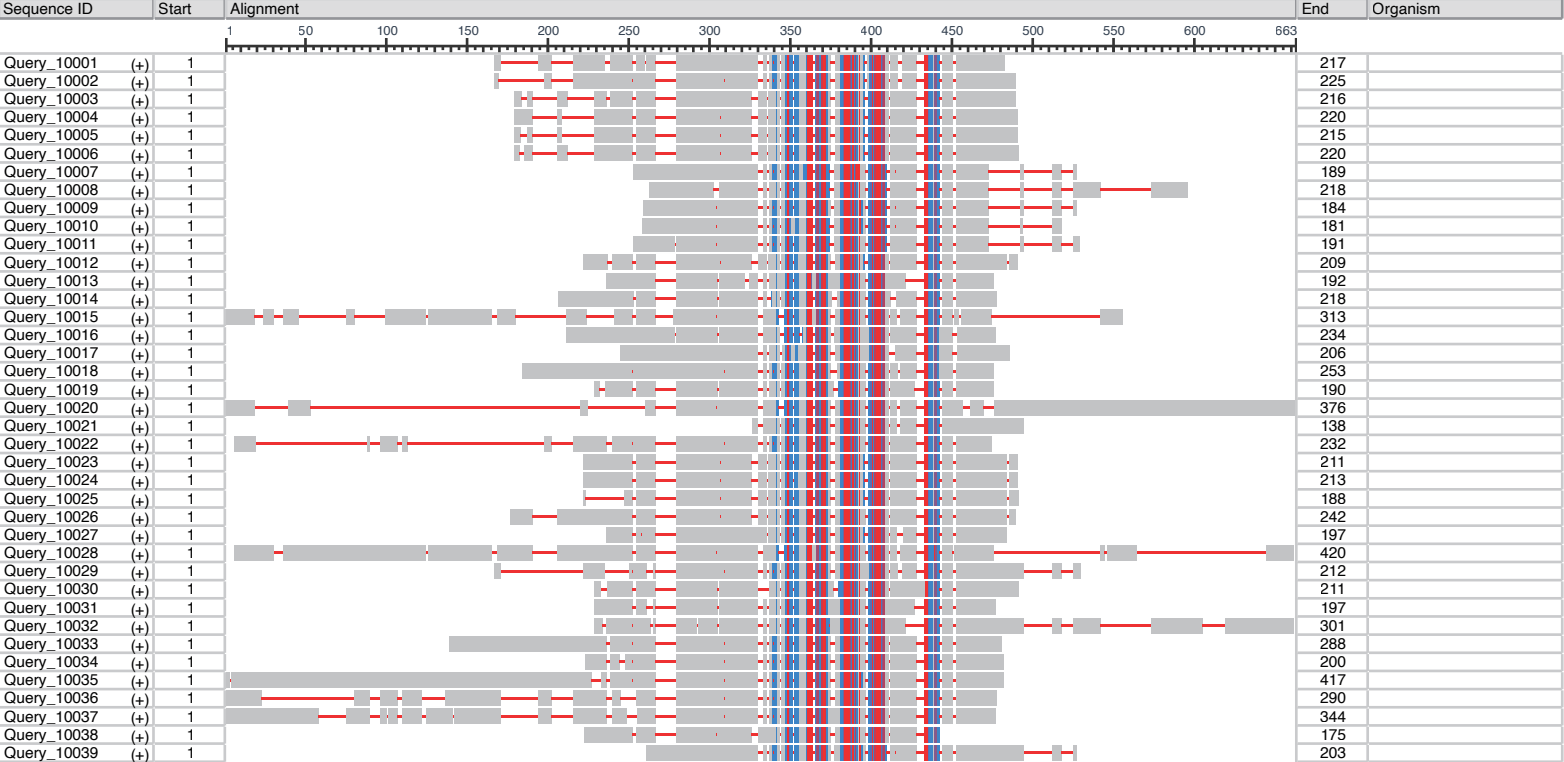

Supplement: Supplementary file 1 — Additional file 1: Figure S1. Expression profiles of UVC exposed R. varieornatus. Gene expression profiles of R. varieornatus specimens exposed to 2.5kJ/m2 UVC shown as a heatmap. TPM values were Z-scaled to between samples. Expression and sample profiles were clustered with by Ward method based on Spearman correlation and the cluster number is indicated on the right side of the heatmap (Cluster numbers from one to eight). Samples are indicated in the following format: Short or long, time point, replicate number. Figure S2. Phylogenetic analysis OG0000230 orthologs within Tardigrada. Phylogenetic tree of tardigrade OG0000230 orthologs and bacterial orthologs. Amino acid sequences were aligned with Mafft, and the phylogenetic tree was constructed with FastTree with 1000 bootstraps (-boot, -gamma). CAHS genes were used as an out-group. Figure S3. Multiple alignment of R. varieornatus and H. exemplaris OG0000231 orthologs. The amino acid sequences of OG0000231 orthologs were submitted to multiple alignment by constraint-based multiple alignment tool from NCBI. Figure S4. Metal-binding property and structural detail of g12777 globular domain. [a,b] The crystal structures of catalytic domain of g12777 complexed with Mn2+ [a] andZn2+ [b]. All crystallographically observed metal ion binding sites are indicated. [c] Topology diagram of g12777 catalytic domain. [d] Close-up view of the Zn2+-binding site. The residues comprising binding site 1(D92, D98, D161, and D163) and the disulfide bond (C91 and C97) are indicated. [e] Conservation of residues. All OG0000231 orthologs were submitted to MEME search. [f] Conserved resides of g12777. The conserved resides are colored according to the bit scores obtained from MEME analysis. [g] Ca2+and Zn2+ ion binding affinity measured by isothermal titration calorimetry. The upper two panels indicate the raw data, while the bottom two panel represent the integrated heat values corrected for the heat of dilution and fit to a one-site bindin [file 12864_2022_8642_MOESM1_ESM.zip › fig_S3.pdf]

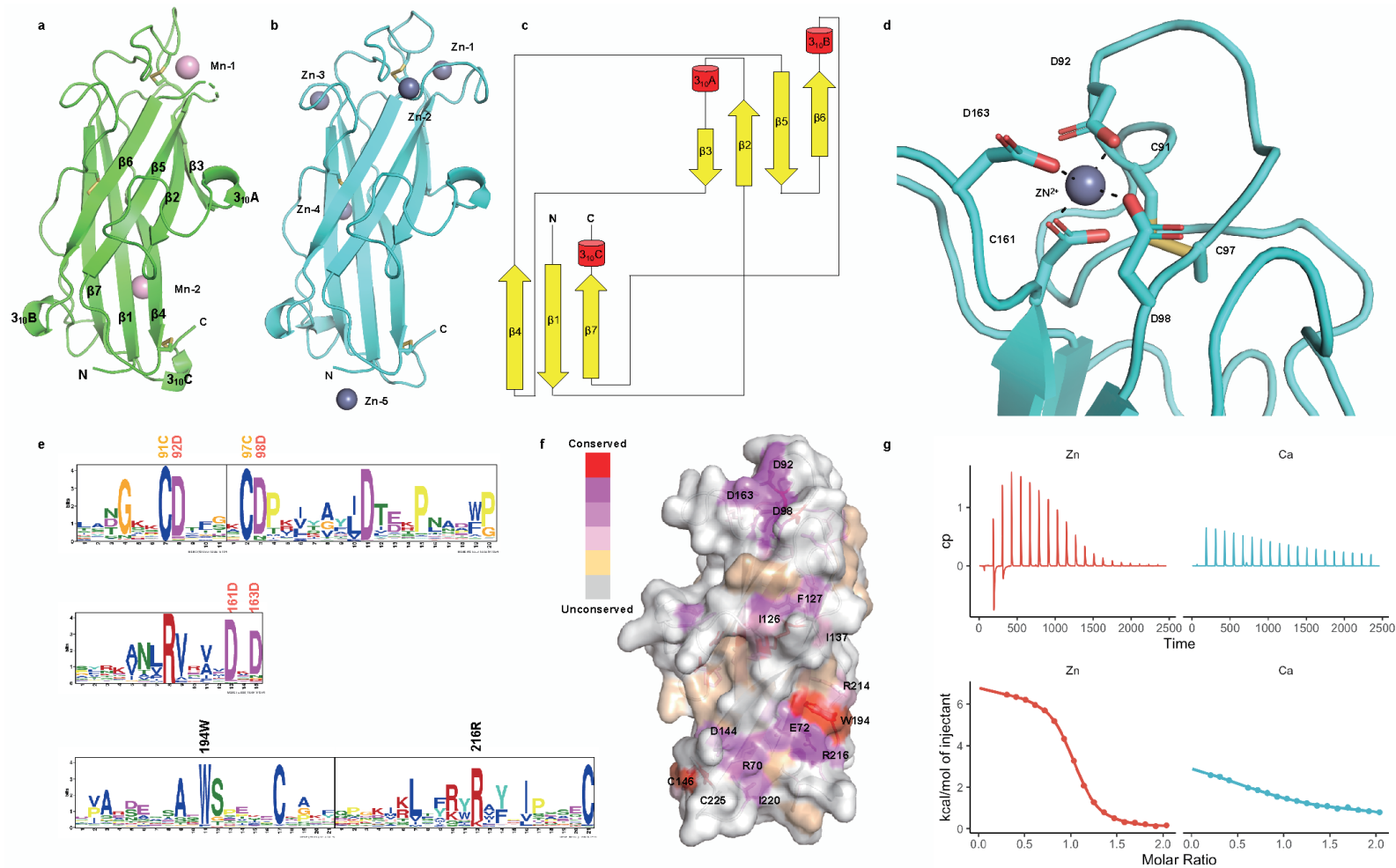

Supplement: Supplementary file 1 — Additional file 1: Figure S1. Expression profiles of UVC exposed R. varieornatus. Gene expression profiles of R. varieornatus specimens exposed to 2.5kJ/m2 UVC shown as a heatmap. TPM values were Z-scaled to between samples. Expression and sample profiles were clustered with by Ward method based on Spearman correlation and the cluster number is indicated on the right side of the heatmap (Cluster numbers from one to eight). Samples are indicated in the following format: Short or long, time point, replicate number. Figure S2. Phylogenetic analysis OG0000230 orthologs within Tardigrada. Phylogenetic tree of tardigrade OG0000230 orthologs and bacterial orthologs. Amino acid sequences were aligned with Mafft, and the phylogenetic tree was constructed with FastTree with 1000 bootstraps (-boot, -gamma). CAHS genes were used as an out-group. Figure S3. Multiple alignment of R. varieornatus and H. exemplaris OG0000231 orthologs. The amino acid sequences of OG0000231 orthologs were submitted to multiple alignment by constraint-based multiple alignment tool from NCBI. Figure S4. Metal-binding property and structural detail of g12777 globular domain. [a,b] The crystal structures of catalytic domain of g12777 complexed with Mn2+ [a] andZn2+ [b]. All crystallographically observed metal ion binding sites are indicated. [c] Topology diagram of g12777 catalytic domain. [d] Close-up view of the Zn2+-binding site. The residues comprising binding site 1(D92, D98, D161, and D163) and the disulfide bond (C91 and C97) are indicated. [e] Conservation of residues. All OG0000231 orthologs were submitted to MEME search. [f] Conserved resides of g12777. The conserved resides are colored according to the bit scores obtained from MEME analysis. [g] Ca2+and Zn2+ ion binding affinity measured by isothermal titration calorimetry. The upper two panels indicate the raw data, while the bottom two panel represent the integrated heat values corrected for the heat of dilution and fit to a one-site bindin [file 12864_2022_8642_MOESM1_ESM.zip › fig_S4.pdf]

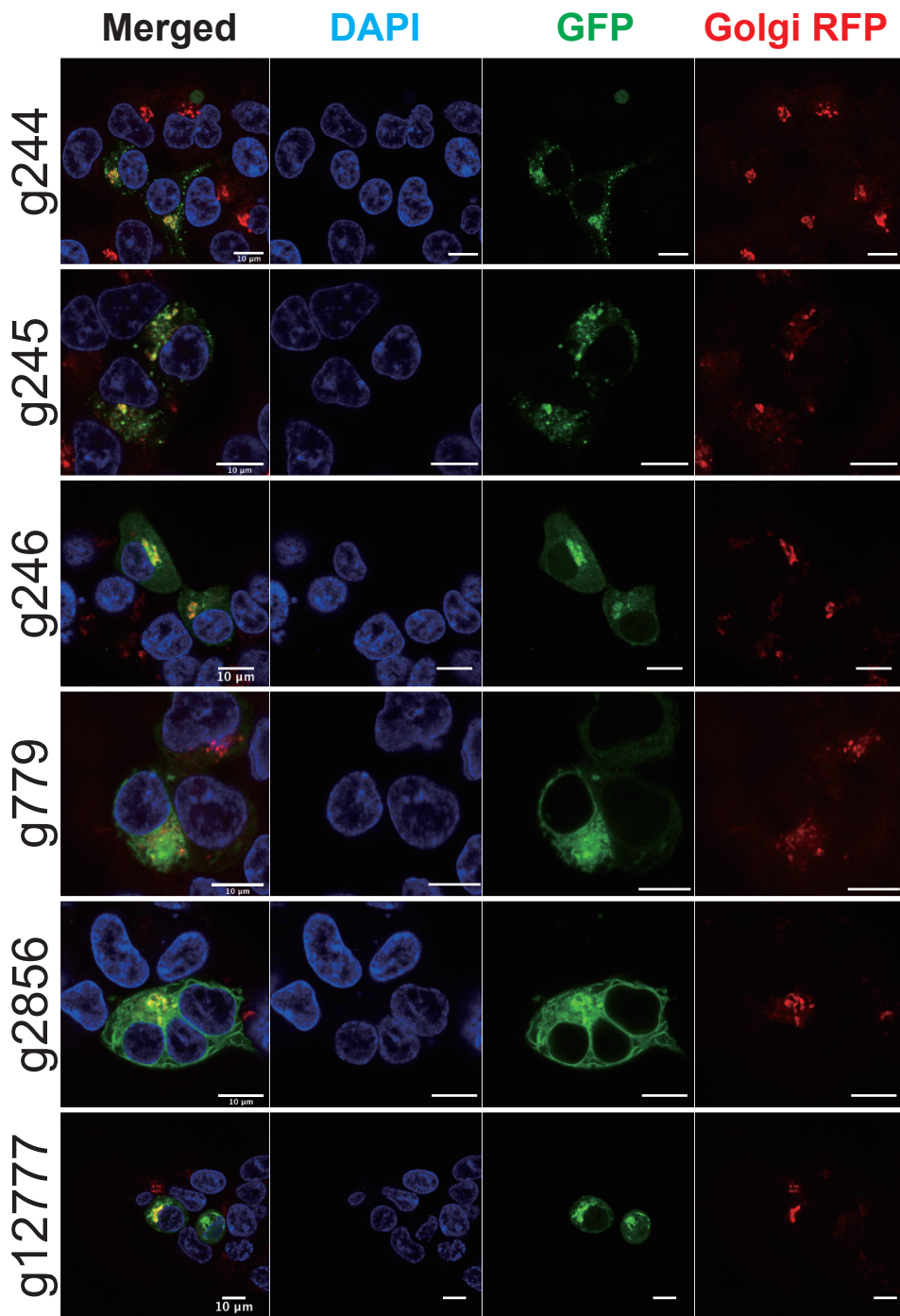

Supplement: Supplementary file 1 — Additional file 1: Figure S1. Expression profiles of UVC exposed R. varieornatus. Gene expression profiles of R. varieornatus specimens exposed to 2.5kJ/m2 UVC shown as a heatmap. TPM values were Z-scaled to between samples. Expression and sample profiles were clustered with by Ward method based on Spearman correlation and the cluster number is indicated on the right side of the heatmap (Cluster numbers from one to eight). Samples are indicated in the following format: Short or long, time point, replicate number. Figure S2. Phylogenetic analysis OG0000230 orthologs within Tardigrada. Phylogenetic tree of tardigrade OG0000230 orthologs and bacterial orthologs. Amino acid sequences were aligned with Mafft, and the phylogenetic tree was constructed with FastTree with 1000 bootstraps (-boot, -gamma). CAHS genes were used as an out-group. Figure S3. Multiple alignment of R. varieornatus and H. exemplaris OG0000231 orthologs. The amino acid sequences of OG0000231 orthologs were submitted to multiple alignment by constraint-based multiple alignment tool from NCBI. Figure S4. Metal-binding property and structural detail of g12777 globular domain. [a,b] The crystal structures of catalytic domain of g12777 complexed with Mn2+ [a] andZn2+ [b]. All crystallographically observed metal ion binding sites are indicated. [c] Topology diagram of g12777 catalytic domain. [d] Close-up view of the Zn2+-binding site. The residues comprising binding site 1(D92, D98, D161, and D163) and the disulfide bond (C91 and C97) are indicated. [e] Conservation of residues. All OG0000231 orthologs were submitted to MEME search. [f] Conserved resides of g12777. The conserved resides are colored according to the bit scores obtained from MEME analysis. [g] Ca2+and Zn2+ ion binding affinity measured by isothermal titration calorimetry. The upper two panels indicate the raw data, while the bottom two panel represent the integrated heat values corrected for the heat of dilution and fit to a one-site bindin [file 12864_2022_8642_MOESM1_ESM.zip › fig_S5.pdf]

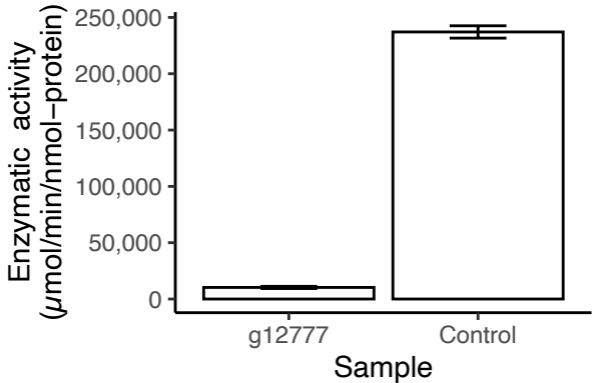

Supplement: Supplementary file 1 — Additional file 1: Figure S1. Expression profiles of UVC exposed R. varieornatus. Gene expression profiles of R. varieornatus specimens exposed to 2.5kJ/m2 UVC shown as a heatmap. TPM values were Z-scaled to between samples. Expression and sample profiles were clustered with by Ward method based on Spearman correlation and the cluster number is indicated on the right side of the heatmap (Cluster numbers from one to eight). Samples are indicated in the following format: Short or long, time point, replicate number. Figure S2. Phylogenetic analysis OG0000230 orthologs within Tardigrada. Phylogenetic tree of tardigrade OG0000230 orthologs and bacterial orthologs. Amino acid sequences were aligned with Mafft, and the phylogenetic tree was constructed with FastTree with 1000 bootstraps (-boot, -gamma). CAHS genes were used as an out-group. Figure S3. Multiple alignment of R. varieornatus and H. exemplaris OG0000231 orthologs. The amino acid sequences of OG0000231 orthologs were submitted to multiple alignment by constraint-based multiple alignment tool from NCBI. Figure S4. Metal-binding property and structural detail of g12777 globular domain. [a,b] The crystal structures of catalytic domain of g12777 complexed with Mn2+ [a] andZn2+ [b]. All crystallographically observed metal ion binding sites are indicated. [c] Topology diagram of g12777 catalytic domain. [d] Close-up view of the Zn2+-binding site. The residues comprising binding site 1(D92, D98, D161, and D163) and the disulfide bond (C91 and C97) are indicated. [e] Conservation of residues. All OG0000231 orthologs were submitted to MEME search. [f] Conserved resides of g12777. The conserved resides are colored according to the bit scores obtained from MEME analysis. [g] Ca2+and Zn2+ ion binding affinity measured by isothermal titration calorimetry. The upper two panels indicate the raw data, while the bottom two panel represent the integrated heat values corrected for the heat of dilution and fit to a one-site bindin [file 12864_2022_8642_MOESM1_ESM.zip › fig_S6.pdf]
